# Effects of “Bacuri” Seed Butter (*Platonia insignis* Mart.) on Metabolic Parameters in Hamsters with Diet-Induced Hypercholesterolemia

**DOI:** 10.1155/2021/5584965

**Published:** 2021-12-06

**Authors:** Geovanni de Morais Lima, Ana Karolinne da Silva Brito, Luciana Melo de Farias, Lays Arnaud Rosal Lopes Rodrigues, Cristian Francisco de Carvalho Pereira, Simone Kelly Rodrigues Lima, Karoline de Macedo Gonçalves Frota, Márcia dos Santos Rizzo, Paulo Humberto Moreira Nunes, Massimo Lucarini, Alessandra Durazzo, Daniel Dias Rufino Arcanjo, Maria do Carmo de Carvalho e Martins

**Affiliations:** ^1^Departamento de Biofísica e Fisiologia, Universidade Federal do Piauí, CEP 64049-550, Teresina, PI, Brazil; ^2^Departamento de Nutrição, Universidade Federal do Piauí, CEP 64049-550, Teresina, PI, Brazil; ^3^Departamento de Morfologia, Universidade Federal do Piauí, CEP 64049-550, Teresina, PI, Brazil; ^4^Departamento de Educação, Instituto Federal de Educação, Ciência e Tecnologia do Maranhão, CEP 65700-000, Bacabal, MA, Brazil; ^5^CREA-Research Centre for Food and Nutrition, Via Ardeatina 546, 00178 Rome, Italy

## Abstract

This study aimed to evaluate the effects of the treatment with bacuri seed butter (BB) on body weight, growth, body mass index, lipid profile, atherosclerotic indices, and liver function in dyslipidemic hamsters. Freshly weaned, male hamsters were divided into four groups: (1) normal group (NG)—maintained with standard chow (AIN-93G); (2) dyslipidemia group (DG)—maintained with hyperlipidemic chow (AIN-93G modified) throughout the follow-up period; (3) bacuri seed butter 25 mg/kg/day (BB-25); and (4) bacuri seed butter 50 mg/kg/day (BB-50). BB groups (25 and 50 mg/kg/day) were also maintained with hyperlipidemic chow throughout the follow-up period, and the treatment started after 21 days receiving a hyperlipidemic diet to induce hypercholesterolemia and maintained for 28 days. No significant differences in triglycerides and total cholesterol were observed for BB-25 and BB-50 groups when compared with NG and DG groups. On the contrary, BB-25 and BB-50 induced both increase of HDL-c (51.40 ± 1.69 and 51.00 ± 2.34, respectively) and decrease of LDL-c (103.80 ± 6.87 and 100.50 ± 3.95, respectively) when compared with DG (41.00 ± 2.94 and 132.70 ± 9.41, respectively). In addition, BB promoted a reduction in the risk of atherosclerotic disease by decreasing (*p* < 0.05) the atherogenic index, coronary artery risk index, and LDL/CT ratio (*p* < 0.05) and increasing HDL/CT ratio. On the contrary, no changes were observed in total cholesterol and triglyceride levels or in body weight, growth, body mass index, or liver function parameters. Thus, bacuri seed butter at doses of 25 and 50 mg/kg/day has positive repercussions on the lipid profile, more precisely on plasma HDL-c and LDL-c, and additionally promotes reduction in the risk of atherosclerosis in hamsters.

## 1. Introduction

Cardiovascular diseases (CVDs) continue to be a major cause of disability and mortality in developed and developing countries. About 45% of all deaths from chronic noncommunicable diseases (NCDs) in the world are caused by cardiovascular diseases [1–3], and in low- and middle-income countries, they account for 88% of premature deaths [4]. Atherosclerotic disease is characterized by lipid accumulation and formation of atheromatous plaques within the endothelium, with consequent impairment of the elastic capacity of the smooth muscle tissue. In this context, dyslipidemia, as a result of hypertriglyceridemia and hypercholesterolemia [5] with elevated levels of low-density lipoprotein cholesterol (LDL-c) and reduced levels of high-density lipoprotein cholesterol (HDL-c), represents a key factor for its development [6].

Pharmacological therapy for the treatment of dyslipidemia is based on the use of statins, resins, and ezetimibe, among others, which help to regulate serum cholesterol levels, reducing the synthesis of cholesterol in the liver and its intestinal absorption [6, 7]. On the contrary, the use of these drugs is limited because of their adverse effects, among them, myalgia, increased hepatic transaminases, and changes in intestinal motility, such as constipation or diarrhea [8, 9]. It is worth mentioning the systematic review for the 2020 US Department of Veterans Affairs and US Department of Defense Guidelines for the management of dyslipidemia published in *Annals of Internal Medicine* by Reston et al. [10]: even if the strength of evidence for most interventions was low or very low, intensified patient care and rechallenging with the same or a different statin (or a lower dose) appear to represent favorable options for improving statin adherence.

In this context, the use of medicinal plants and their products in the treatment of dyslipidemia has been increasing because natural products present a lower cost when compared to synthetic, as well as their obtention is suitable [11]. Generally, medicinal plants and herbs are widely being used as sources of nutraceutical active compounds for the management of several types of diseases [12, 13]. The nutraceutical approach to dyslipidemia has been described in different papers as a possible alternative to the conventional drug-based therapy and/or adjuvant therapy using promising natural agents [13–15].

In addition, it is noteworthy that dietary interventions, especially those that provide a large intake of foods with functional properties, can delay or reduce the risk of the development and progression of chronic diseases by modulation of body physiological functions [16]. Studies have shown that plant foods and their derived extracts, for example, can act on a variety of intermediate markers of cardiometabolic risk, including blood pressure, glucose-insulin homeostasis, blood lipids and lipoproteins, endothelial function, inflammation, and oxidative stress. These products have gained increasing notoriety in the last decade due to emerging evidence of their role in important pathways, modulating responses capable of promoting cardiovascular health benefits [3, 17].

“Bacurizeiro” (*Platonia insignis* Mart.) is a plant typical of Cerrado, belonging to the family Clusiaceae and to the genus *Platonia* [18, 19], and has been used in folk medicine in the treatment of diarrhea [20], wounds, and other skin conditions [21]. Some studies have investigated different biological activities of bacuri, and they are identified as antioxidant [22, 23], anti-inflammatory [24], wound healing [25, 26], anticonvulsant [27, 28], antileishmanial [18, 29, 30], and immunomodulatory [31], among other effects. Moreover, formulation of bacuri-based functional products is being investigated [24, 26, 32].

There have been no studies to date on the effects of bacuri seed butter (*Platonia insignis* Mart.) on experimental dyslipidemia, and then considering its wide pharmacological and nutraceutical potentials, the present work aimed to investigate its effects in hamsters with diet-induced hypercholesterolemia.

## 2. Materials and Methods

### 2.1. Materials

Bacuri seed butter (*Platonia insignis* Mart.) was supplied by Amazon Oil Indústria e Comércio Ltda (Ananindeua, PA, Brazil). This butter is cold extracted from wild species that grow naturally in the Amazon rainforest, sustainably extracted without using pesticides and fertilizers. No preservatives, additives, or any other chemical substances are added (see https://www.amazonoil.com.br/pt/perfil/).

### 2.2. Animals and Diets

Freshly weaned, male hamsters (*Mesocricetus auratus*) were obtained from AniLab Laboratory Animals Ltd. (Paulínia, SP, Brazil). Hamsters were kept in individual cages under controlled conditions: temperature 24 ± 2°C; 12 h light/dark cycle; humidity (55%); water and chow *ad libitum*. After twenty days of adaptation, the animals were divided into four groups: (1) normal group (NG; *n* = 8)—maintained with standard chow (AIN-93G); (2) dyslipidemia group (DG; *n* = 8)—maintained with hyperlipidemic chow (AIN-93G modified) throughout the follow-up period; (3) bacuri seed butter 25 mg/kg/day (BB-25; *n* = 10); and (4) bacuri seed butter 50 mg/kg/day (BB-50; *n* = 10). BB groups (25 and 50 mg/kg/day) were also maintained with hyperlipidemic chow throughout the follow-up period, and the treatment started after 21 days receiving a hyperlipidemic diet to induce hypercholesterolemia. The animals were kept with standard or experimental (modified) chows produced according to the American Institute of Nutrition (AIN) 93G [33]. The composition of experimental diets is listed in Table 1.

Bacuri butter (25 or 50 mg/kg/day) was administered orally once daily, dissolved in 0.1% Tween 80 in distilled water (5 ml/kg volume). Normal and dyslipidemia groups received daily vehicle volume (0.1% Tween 80 in distilled water). After 28 days of treatment, the animals were euthanized by an overdose of sodium thiopental (100 mg/kg) mixed with lidocaine (10 mg/mL) i.p., and blood samples were collected.

Food intake was monitored every two days and body weight every three days. The nasoanal length was determined on the first day of induction of hypercholesterolemia, on the first day of treatment, and on the day of euthanasia. From these data, the Lee index was calculated by the following formula: $\text{Lee}=\left(\sqrt[{3}]{\text{weight}}\;÷\text{NL}\right)\times 10,000$, where “NL” stands for nasoanal length, while the body mass index (BMI) was obtained dividing weight by the square of nasoanal length.

All procedures performed were approved by the Ethics Committee on the Use of Animals of the Federal University of Piauí (CEUA/UFPI) (authorization no. 197/16).

For analyses of the centesimal composition of the standard and hypercholesterolemic diets (Table 2), the moisture, ash, lipid, and protein contents were determined according to the method described by the Association of Official Analytical Chemists (AOAC) [34]. The total carbohydrate content of the samples was estimated by difference: [100 − (moisture + ash + protein + lipids)]. Data were expressed in g/100 g of dry matter (energy conversion factors: protein 17 kJ/g; fat 37 kJ/g; total carbohydrates 17 kJ).

### 2.3. Lipid Profile and Liver Function

Triglyceride (TG), total cholesterol (TC), high-density lipoprotein cholesterol (HDL-c), alanine transaminase (ALT), aspartate aminotransferase (AST), and alkaline phosphatase (ALK) levels were analyzed using diagnostic kits, purchased from Labtest (São Paulo, BR), according to the manufacturer's specifications and Labmax Plenno Automated Chemistry Analyzer. Low-density lipoprotein cholesterol (LDL-c) values were obtained from the Friedewald formula: LDL − c=total cholesterol − (HDL cholesterol+triglyceride ÷ 5) [35].

### 2.4. Atherosclerosis Indices

The coronary artery risk index (CRI) was calculated by dividing plasma levels of LDL-c by the HDL-c levels, according to Draper et al. [36]. Atherogenic index (AI) was calculated according to Roth et al. [1] by dividing the triglyceride levels by the HDL-c levels.

The HDL/TC ratio was calculated by the following formula: HDL − c/TC ratio=HDL − c ÷ TC, while the LDL-c/TC ratio was calculated by LDL − c/TC ratio=LDL − c ÷ TC, according to Lee et al. [37].

### 2.5. Statistical Analyses

The values were represented as mean ± standard deviation of the mean. Statistical analysis was performed by one-way ANOVA followed by Tukey's posttest for multiple comparisons. The level of significance was set at *p* < 0.05.

## 3. Results and Discussion

Here, main findings are highlighted and contextualized as follows: (i) food consumption, body weight, growth, and body mass index; (ii) lipid profile of hamsters with diet-induced hypercholesterolemia; (iii) cardiovascular risk indexes of hamsters with diet-induced hypercholesterolemia; (iv) liver function of hamsters with diet-induced hypercholesterolemia.

### 3.1. Food Consumption, Body Weight, Growth, and Body Mass Index

Hyperlipidemic diet has caused a significant reduction (*p* < 0.05) in food intake in dyslipidemic animals when compared to normal animals receiving standard chow (Table 3). Despite this, hypercholesterolemic hamsters did not differ (*p* > 0.05) in relation to body weight, length, and body mass indices (BMI and Lee) at the end of the experimental period. According to Kretschmer et al. [38], animals fed a diet high in fat/carbohydrates and were able to detect the energy content of the food and compensate for this with a lower intake, which may explain the lower consumption observed in groups BB and DG. Although there is a discrepancy between the results reported in the literature [39–41], there is a consensus that this difference in consumption may be strongly related to the duration of the animals' maintenance on that diet and the time of initiation of treatment [38, 41].

These results can be attributed to the duration of the experimental period since the total time of the hyperlipidemic diet was only 7 weeks and other studies used protocols of longer duration to induce obesity in hamsters, with 12 to 16 weeks, and despite this, there were no differences in relation to weight gain among groups, probably due to the higher amount of kcal/g in hyperlipidemic diet [42–44].

### 3.2. Lipid Profile of Hamsters with Diet-Induced Hypercholesterolemia

Hyperlipidemia induced by high-fat diet in rodents is a widely used model for the evaluation of compounds with alleged hypolipidemic effect [45]. In this context, coconut oil and cholesterol were used as sources of lipids for induction of hypercholesterolemia in this study. Coconut oil is rich in saturated fatty acids and produces elevation of triglyceride, total cholesterol, and LDL cholesterol levels [46, 47]. Similarly, cholesterol intake promotes elevation of total cholesterol (TC) levels, contributing for induction of dyslipidemia [48, 49].

The lipid metabolism of *Mesocricetus auratus* makes this species one of the best models for the study of dyslipidemia due to the similarity to that of humans, in which the transport of cholesterol in blood occurs mainly in the form of LDL cholesterol; and the elevation of dietary lipid intake is followed by an increase in triglyceride levels, unlike other rodents [50–53].

**Figure 1:**
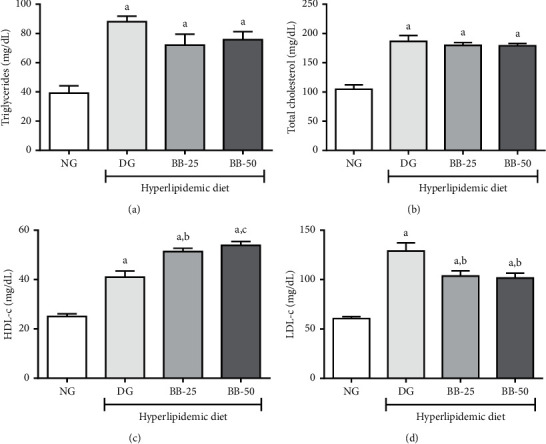
Triglycerides, total cholesterol, HDL-c, and LDL-c serum levels in hamsters (*Mesocricetus auratus*) after 28 days of treatment with bacuri seed butter (25 or 50 mg/kg/day). NG: normal group; DG: dyslipidemia group; BB-25: bacuri seed butter 25 mg/kg/day; BB-50: bacuri seed butter 50 mg/kg/day. ^a^*p* < 0.01 in relation to NG; ^b^*p* < 0.05 in relation to DG; ^c^*p* < 0.01 in relation to DG according to Tukey's *post hoc* test.

Thus, the effects of bacuri butter on the lipid profile of hypercholesterolemic hamsters were evaluated (Figure 1). It was observed that hyperlipidemic diet promoted a significant increase (*p* < 0.05) in the levels of triglycerides, total cholesterol, HDL-c, and LDL-c. In addition, BB was shown to have an atheroprotective effect by increasing (*p* < 0.05) HDL-c levels and reducing (*p* < 0.05) LDL-c levels in BB-treated animals when compared to the dyslipidemic group.

HDL cholesterol is initially synthesized in the liver in the form of apolipoprotein A1 and transferred to the bloodstream where it binds to phospholipids and cholesterol, as well as promotes efflux of cholesterol stored in cells and subsequently carries cholesterol to the liver to be excreted in the feces [54]. The reduction of LDL cholesterol is associated with the reduction of cardiovascular risk due to its ability to cross the vascular endothelium and accumulate, undergoing oxidation and initiating the formation of atherosclerotic lesions [55]. In this sense, the risk of atherogenicity was assessed using cardiovascular risk indices of animals treated with bacuri seed butter.

The oil extracted from the bacuri seed predominantly contains saturated fatty acids, such as palmitic acid, and monounsaturated acids, such as oleic and palmitoleic acids [56]. Saturated fatty acids' intake leads to an increase in total cholesterol and LDL cholesterol levels by increasing the synthesis of hepatic cholesterol and by reducing the activity of LDL receptors, while unsaturated (poly- or monounsaturated) acids promote the increase in the activity and in the amount of LDL receptors, as well as in its mRNA, thus increasing its turnover [57]. In this sense, it was suggested that the effects of BB on the lipid profile were at least in part due to its content of unsaturated fatty acids.

Bacuri is rich in secondary metabolites, especially xanthones and chemical precursors thereof, such as polyisoprenylated benzophenones [22, 29]. Miura et al. [58] investigated the effects of mangiferin, a xanthone, on mice with hypercholesterolemia induced by high cholesterol intake and observed a reduction in total cholesterol levels in treated animals. In another study, Bao et al. [59] demonstrated that dimethoxyxanthone and trimethoxyxanthone improved lipid metabolism in obese rats induced by a high-fructose diet by reducing total cholesterol, triglyceride, and LDL cholesterol levels and raising HDL cholesterol levels. In this context, xanthones may have contributed to BB effects on the lipid profile of hypercholesterolemic hamsters.

### 3.3. Cardiovascular Risk Indexes of Hamsters with Diet-Induced Hypercholesterolemia

Additionally, BB was found to reduce the risk of cardiovascular disease by promoting reduction (*p* < 0.05) in atherogenic and coronary artery risks (Table 4). These results are worth noting since high AI values correlate with elevated blood pressure and metabolic dysfunctions and diseases, such as hyperinsulinemia [60].

Furthermore, untreated hypercholesterolemic hamsters were found to have lower HDL/TC and higher LDL/TC ratios, which indicate a higher risk of atherosclerosis severity in the dyslipidemia group when compared to the groups treated with BB. Similarly, but using different animal species, Basu et al. [61] observed that *Hippophae rhamnoides* seed oil, popularly known as common sea buckthorn, promoted an increase in the HDL/TC ratio and reduced the risk of atherosclerosis in hypercholesterolemic rabbits after 30 days of supplementation with 1% cholesterol.

### 3.4. Liver Function of Hamsters with Diet-Induced Hypercholesterolemia

Afterwards, it was verified that the ingestion of hyperlipidemic diet did not cause change of the levels of AST, ALT, and ALK at the end of the experimental period (Table 5). Likewise, BB did not produce changes in liver function, which indicates that its administration does not produce systemic toxicity. Similarly, however, using a hypercholesterolemic diet composed of a standard diet plus 1% cholesterol, Martinello et al. [62] observed that high-fat intake, for 10 weeks, did not promote an increase on levels of AST and ALT in hamsters fed by a high-fat diet. On the contrary, Lai et al. [63], using a hypercholesterolemic diet composed of standard diet supplemented with 11.5% coconut oil, 11.5% corn oil, and 1% cholesterol, observed that hypercholesterolemic hamsters with dyslipidemia induced for 12 weeks presented increased levels of AST and ALT compared to the normal group. Also, in a different way, Yang et al. [64] found that hamsters fed by a high-fat diet of 94.9% of standard feed, 5% of Ching-Shan oil, and 0.1% of cholesterol showed a significant increase in serum levels of AST and ALT.

This study has a pioneering character in relation to the use of bacuri butter in dyslipidemia models, and although bacuri is a monotype fruit of Amazonian origin, the Clusiaceae family encompasses approximately 1000 species belonging to 47 genera [65, 66], which leads to the possibility of further research being conducted with different species distributed in the most diverse parts of the world.

Bacuri butter is industrially obtained through cold pressing of the fruit seeds, and it is worth noting that, although pre- and postharvest factors can influence the composition of the fruits, the compositional analyses available in the literature show that no important nutritional variations occur as a result of these factors, although the physical-chemical characteristics may be influenced by the industrial treatment received during the process of obtaining the final product, which may originate virgin or clarified butter [67–69].

Furthermore, future research using longer clinical trial protocols will be needed for a better understanding of outcomes related to cardiovascular health in order to develop safe guidelines for an effective indication as a preventive or auxiliary agent in the treatment of dyslipidemia in humans.

## 4. Conclusion

Bacuri seed butter, at the doses and schedule used in this study, has positive repercussions on the lipid profile, more precisely on the fractions of HDL-c and LDL-c, and additionally promotes reduction in the risk of atherosclerosis in hamsters. Furthermore, BB did not produce deleterious effects on liver enzyme activity, weight gain, growth, body mass indices, or food intake in hamsters fed by a high-fat diet. Future directions could be the application of nanotechnologies to an improvement of functional properties of the bacuri component in the perspective of nanonutraceutical science [70].

## Figures and Tables

**Table 1:** Composition of experimental diets (g/kg).

Ingredients	AIN-93G	AIN-93G modified
Casein	200.0	221.0
L-cystine	3.0	3.0
Corn starch	397.5	427.5
Dextrinized corn starch	132.0	—
Sucrose	100.0	50.0
Fiber	50.0	100.0
Soybean oil	70.0	20.0
Coconut oil	—	130.0
Cholesterol	—	1.0
*tert*-Butylhydroquinone	0.014	0.024
Mineral mix (S10022G)	35.0	35.0
Vitamin mix (V10037)	10.0	10.0
Choline bitartrate	2.5	2.5

**Table 2:** Centesimal composition of rations (g/100 g dry matter).

Components	Standard	Hypercholesterolemic
Moisture (%)	10.20 ± 0.10	6.85 ± 0.20^*∗*^
Ash (%)	7.80 ± 0.10	4.80 ± 0.20^*∗*^
Lipids (%)	3.30 ± 0.10	14.61 ± 0.10^*∗*^
Protein (%)	20.90 ± 0.40	19.06 ± 1.00
Total carbohydrate (%)	57.80 ± 0.30^*#*^	54.68 ± 0.80^*#*^
TEV (kJ·g^−1^)	14.42	17.85

Mean ± standard deviation. TEV: total energy value. #Carbohydrate calculated by difference, including fibers. ^*∗*^*p* < 0.05 when compared with normolipidemic feed, Student's *t*-test.

**Table 3:** Food intake, body weight, and nasoanal length indices in hamsters (*Mesocricetus auratus*) after 28 days of treatment with bacuri seed butter (25 or 50 mg/kg/day).

Parameters	Groups (mean ± SEM)			
	NG	DG	BB-25	BB-50
Daily food intake (g)	9.42 ± 0.21	7.49 ± 0.18a	7.47 ± 0.38a	7.44 ± 0.26^a^
Initial body weight (g)	114.37 ± 4.08	113.25 ± 6.43	112.44 ± 5.24	112.87 ± 4.14
Final body weight (g)	132.12 ± 4.52	134.87 ± 4.41	132 ± 5.36	129.25 ± 5.60
Weight gain (g)	83.57 ± 4.67	74.29 ± 3.83	73.71 ± 2.61	79.11 ± 4.46
Initial nasoanal length (cm)	15.24 ± 0.26	15.00 ± 0.35	14.66 ± 0.88	15.70 ± 0.25
Final nasoanal length (cm)	16.75 ± 0.43	17.12 ± 0.41	17.2 ± 0.33	17.35 ± 0.14
Lee index	3,073.00 ± 33.33	3,020.00 ± 102.70	3,016.00 ± 32.15	2,970.00 ± 37.87
BMI	0.48 ± 0.01	0.46 ± 0.02	0.46 ± 0.01	0.43 ± 0.01

NG: normal group; DG: dyslipidemia group; BB-25: bacuri seed butter 25 mg/kg/day; BB-50: bacuri seed butter 50 mg/kg/day; ^a^*p* < 0.05 in relation to NG according to Tukey's *post hoc* test.

**Table 4:** Effects of bacuri seed butter (25 or 50 mg/kg/day) on the atherogenic index (AI), coronary artery risk index (CRI), HDL/TC ratio, and LDL/TC ratio in hamsters (*Mesocricetus auratus*) with diet-induced hypercholesterolemia.

Groups	Parameters (mean ± SEM)			
	AI	CRI	HDL/TC ratio	LDL/TC ratio
NG	1.47 ± 0.16	2.42 ± 0.19	0.238 ± 0.01	0.578 ± 0.04
DG	2.14 ± 0.16^a^	3.14 ± 0.19^a^	0.219 ± 0.02	0.691 ± 0.07^a^
BB-25	1.4 ± 0.16^b^	2.01 ± 0.11^b^	0.285 ± 0.01^a.b^	0.576 ± 0.03^b^
BB-50	1.4 ± 0.15^b^	1.88 ± 0.09^a.b^	0.300 ± 0.01^a.b^	0.568 ± 0.01^b^

NG: normal group; DG: dyslipidemia group; BB-25: bacuri seed butter 25 mg/kg/day; BB-50: bacuri seed butter 50 mg/kg/day; AI: atherogenic index; CRI: coronary artery risk index. ^a^*p* < 0.05 in relation to NG. ^b^*p* < 0.05 in relation to DG according to Tukey's *post hoc* test.

**Table 5:** Serum levels of AST, ALT, and ALP (U/L) in hamsters (*Mesocricetus auratus*) after 28 days of treatment with bacuri seed butter (25 or 50 mg/kg/day).

Groups	Parameters (mean ± SEM)		
	AST (U/L)	ALT (U/L)	ALP (U/L)
NG	61.42 ± 5.69	73.66 ± 6.95	267.66 ± 41.97
DG	69.85 ± 14.36	65.50 ± 7.93	363.6 ± 78.83
BB-25	71.87 ± 5.11	68.16 ± 4.94	353.66 ± 53.53
BB-50	80.28 ± 6.28	83.28 ± 4.8	320.62 ± 13.25

NG: normal group; DG: dyslipidemia group; BB-25: bacuri seed butter 25 mg/kg/day; BB-50: bacuri seed butter 50 mg/kg/day; AST: aspartate transaminase; ALT: alanine aminotransferase; ALP: alkaline phosphatase. No significant differences were observed according to Tukey's *post hoc* test.

## Data Availability

The data used to support the findings of this study are available from the corresponding author upon request.

## Abbreviations

AI: Atherogenic index
AIN: American Institute of Nutrition
ALP: Alkaline phosphatase
ALT: Alanine transaminase
AOAC: Association of Official Analytical Chemists
AST: Aspartate aminotransferase
BB: Bacuri seed butter
BMI: Body mass index
CNCDs: Chronic noncommunicable diseases
CRI: Coronary artery risk index
CVD: Cardiovascular disease
DG: Dyslipidemia group
HDL-c: High-density lipoprotein cholesterol
LDL-c: Low-density lipoprotein cholesterol
NG: Normal group
TC: Total cholesterol
TEV: Total energy value
TG: Triglycerides.

## References

[1] Roth G. A., Forouzanfar M. H., Moran A. E. (2015). Demographic and epidemiologic drivers of global cardiovascular mortality. *New England Journal of Medicine*.

[2] Van Bussel E. F., Hoevenaar-Blom M. P., Poortvliet R. K. E. (2020). Predictive value of traditional risk factors for cardiovascular disease in older people: a systematic review. *Preventive Medicine*.

[3] Oliveira G. M. M. d., Brant L. C. C., Polanczyk C. A. (2020). Estatística cardiovascular-brasil 2020. *Arquivos Brasileiros de Cardiologia*.

[4] Nascimento B. R., Brant L. C. C., Oliveira G. M. M. (2018). Cardiovascular disease epidemiology in Portuguese-speaking countries: data from the global burden of disease, 1990 to 2016. *Arquivos Brasileiros de Cardiologia*.

[5] Ramasamy I. (2016). Update on the molecular biology of dyslipidemias. *Clinica Chimica Acta*.

[6] Mach F., Baigent C., Catapano A. L. (2020). ESC scientific document group, 2019 ESC/EAS guidelines for the management of dyslipidaemias: lipid modification to reduce cardiovascular risk: the task force for the management of dyslipidaemias of the European society of cardiology (ESC) and European atherosclerosis society (EAS). *European Heart Journal*.

[7] Magalhães M. E. C., Brandão A. A., Freitads E. V., Pozzan R. B. A. (2004). New perspectives on dislipidemia therapy. *SOCERJ*.

[8] Grundy S. M., Arai H., Barter P. (2014). An international atherosclerosis society position paper: global recommendations for the management of dyslipidemia-full report. *Journal of Clinical Lipidology*.

[9] Bader T. (2010). The myth of statin-induced hepatotoxicity. *American Journal of Gastroenterology*.

[10] Reston J. T., Buelt A., Donahue M. P., Neubauer B., Vagichev E., McShea K. (2020). Interventions to improve statin tolerance and adherence in patients at risk for cardiovascular disease. *Annals of Internal Medicine*.

[11] Simões C. M. O., Auler Mentz L., Schenkel E. P. (1988). *Medicinal Plants in Rio Grande Do Sul*.

[12] Durazzo A., D’Addezio L., Camilli E. (2018). From plant compounds to botanicals and back: a current snapshot. *Molecules*.

[13] Novellino C. E., Federico N. (2017). Via II (2017) Nutraceuticals in hypercholesterolaemia: an overview. *British Journal of Pharmacology*.

[14] Mollazadeh H., Mahdian D., Hosseinzadeh H. (2019). Medicinal plants in treatment of hypertriglyceridemia: a review based on their mechanisms and effectiveness. *Phytomedicine*.

[15] Bahmani M., Mirhoseini M., Shirzad H., Sedighi M., Shahinfard N., Rafieian-Kopaei M. (2015). A review on promising natural agents effective on hyperlipidemia. *Journal of Evidence-Based Complementary & Alternative Medicine*.

[16] Cicero A. F. G., Colletti A. (2017). Food and plant bioactives for reducing cardiometabolic disease: how does the evidence stack up?. *Trends in Food Science & Technology*.

[17] Morand C., Tomás-Barberán F. A. (2019). Contribution of plant food bioactives in promoting health effects of plant foods: why look at interindividual variability?. *European Journal of Nutrition*.

[18] Souza A. C., Alves M. M. M., Brito L. M. (2017). Platonia insignis mart., a Brazilian amazonian plant: the stem barks extract and its main constituent lupeol exert antileishmanial effects involving macrophages activation. *Evidence-based Complementary and Alternative Medicine: ECAM*.

[19] Souza I. G. B., Souza V. A. B., Lima P. S. C. (2013). Molecular characterization of Platonia insignis Mart. (“Bacurizeiro”) using inter simple sequence repeat (ISSR) markers. *Molecular Biology Reports*.

[20] Oliveira Junior S. R., Conceição G. M. (2010). Medicinal plants used in community of brejinho, caxias, maranhão state, Brazil. *Cadernos de Geociencias*.

[21] Agra M. D. F., Silva K. N., Basílio I. J. L. D., Freitas P. F. D., Barbosa-Filho J. M. (2008). Survey of medicinal plants used in the region Northeast of Brazil. *Revista Brasileira de Farmacognosia*.

[22] Da Costa Júnior J. S., De Almeida A. A. C., Costa J. P., das Graças Lopes Citó A. M., Saffi J., Mendes de Freitas R. (2012). Superoxide dismutase and catalase activities in rat hippocampus pretreated with garcinielliptone FC from Platonia insignis. *Pharmacien Biologiste*.

[23] do Nascimento Cavalcante A., Lima L. K. F., Araújo C. M. (2020). Toxicity, cytotoxicity, mutagenicity and in vitro antioxidant models of 2-oleyl-1,3-dipalmitoyl-glycerol isolated from the hexane extract of Platonia insignis MART seeds. *Toxicology Reports*.

[24] Lustosa A. K. M. F., Silva F. V., Silva E. R. S. (2020). Lecithin-based organogel for an industrialized butter from Platonia insignis Mart. seeds and its anti-inflammatory potential: formulation and preclinical studies. *Journal of Global Innovations*.

[25] Mendes M. C. S., Oliveira G. A. L., Lacerda J. S. (2015). Evaluation of the cicatrizant activity of a semisolid pharmaceutical formulation obtained from Platonia insignis Mart. *African Journal of Pharmacy and Pharmacology*.

[26] Lustosa A. K. M. F., Coêlho A. G., Santos A. A. (2021). Topical formulations based on seeds butter from Platonia insignis Mart. for the treatment of injuries related to experimental cutaneous leishmaniasis. *Research, Society and Development*.

[27] Da Costa J. S., De Almeida A. A. C., Tomé A. d. R., Citó A. M. d. G. L., Saffi J., de Freitas R. M. (2011). Evaluation of possible antioxidant and anticonvulsant effects of the ethyl acetate fraction from Platonia insignis Mart. (Bacuri) on epilepsy models. *Epilepsy and Behavior*.

[28] da Costa J. S., Feitosa C. M., das Graças Lopes Citó A. M., de Freitas R. M., Pegas Henrique J. A., Saffi J. (2010). Evaluation of effects of ethanolic extract from Platonia insignis mart, on pilocarpine-induced seizures. *Journal of Biological Sciences*.

[29] Costa Júnior J. S., de Almeida A. A. C., Ferraz A. (2013). Cytotoxic and leishmanicidal properties of garcinielliptone FC, a prenylated benzophenone from Platonia insignis. *Natural Product Research*.

[30] Lustosa A. K. M. F., Bezerra ÉA. B., Rodrigues K. A. (2018). Antileishmanial effect of fruit seeds from Platonia insignis against macrophage-internalized amastigote forms of Leishmania amazonensis. *Rev Cuba Plantas*.

[31] Lustosa A. K. M. F., Arcanjo D. D. R., Ribeiro R. G. (2016). Immunomodulatory and toxicological evaluation of the fruit seeds from Platonia insignis, a native species from Brazilian Amazon Rainforest. *Revista Brasileira de Farmacognosia*.

[32] Silva T. G., Kasemodel M. G. C., Ferreira O. M., Silva R. C. L., Souza C. J. F., Sanjinez‐Argandona E. J. (2020). Addition of Pachira aquatica oil and Platonia insignis almond in cookies: physicochemical and sensorial aspects. *Food Sciences and Nutrition*.

[33] Reeves P. G., Nielsen F. H., Fahey G. C. (1993). AIN-93 purified diets for laboratory rodents: final report of the American institute of nutrition ad hoc writing committee on the reformulation of the AIN-76a rodent diet. *Journal of Nutrition*.

[34] Association of Offccial Analytical Chemists (AOAC) (2016). *Offcial Methods of Analysis of AOAC International*.

[35] Friedewald W. T., Levy R. I., Fredrickson D. S. (1972). Estimation of the concentration of low-density lipoprotein cholesterol in plasma, without use of the preparative ultracentrifuge. *Clinical Chemistry*.

[36] Draper M. W., Flowers D. E., Huster W. J., Neild J. A., Harper K. D., Arnaud C. (1996). A controlled trial of raloxifene (LY139481) HCl: impact on bone turnover and serum lipid profile in healthy postmenopausal women. *Journal of Bone and Mineral Research: The Official Journal of the American Society for Bone and Mineral Research*.

[37] Lee H. S., Lee Y. J., Chung Y. H. (2015). Beneficial effects of red yeast rice on high-fat diet-induced obesity, hyperlipidemia, and fatty liver in mice. *Journal of Medicinal Food*.

[38] Kretschmer B. D., Schelling P., Beier N. (2005). Modulatory role of food, feeding regime and physical exercise on body weight and insulin resistance. *Life Sciences*.

[39] Duarte A. C. G. d. O., Fonseca D. F., Manzoni M. S. J. (2006). Dieta hiperlipídica e capacidade secretória de insulina em ratos. *Revista de Nutrição*.

[40] Oscai L. B., Miller W. C., Arnall D. A. (1987). Effects of dietary sugar and of dietary fat on food intake and body fat content in rats. *Growth*.

[41] Kim E.-M., Welch C. C., Grace M. K., Billington C. J., Levine A. S. (1998). Effects of palatability-induced hyperphagia and food restriction on mRNA levels of neuropeptide-Y in the arcuate nucleus. *Brain Research*.

[42] Décordé K., Teissèdre P. L., Sutra T., Ventura E., Cristol J. P., Rouanet J. M. (2009). Chardonnay grape seed procyanidin extract supplementation prevents high-fat diet-induced obesity in hamsters by improving adipokine imbalance and oxidative stress markers. *Molecular Nutrition & Food Research*.

[43] Suh J.-H., Romain C., González-Barrio R. (2011). Raspberry juice consumption, oxidative stress and reduction of atherosclerosis risk factors in hypercholesterolemic golden Syrian hamsters. *Food & Function*.

[44] Vidé J., Virsolvy A., Romain C. (2015). Dietary silicon-enriched spirulina improves early atherosclerosis markers in hamsters on a high-fat diet. *Nutrition*.

[45] Buettner G. R. (2012). Superoxide dismutase in redox biology: the roles of superoxide and hydrogen peroxide. *Anti-Cancer Agents in Medicinal Chemistry*.

[46] Eyres L., Eyres M. F., Chisholm A., Brown R. C. (2016). Coconut oil consumption and cardiovascular risk factors in humans. *Nutrition Reviews*.

[47] Oda T., Aoe S., Imanishi S., Kanazawa Y., Sanada H., Ayano Y. (1994). Effects of dietary oat, barley, and guar gums on serum and liver lipid concentrations in diet-induced hypertriglyceridemic rats. *Journal of Nutritional Science & Vitaminology*.

[48] Stamler J., Shekelle R. (1988). Dietary cholesterol and human coronary heart disease. The epidemiologic evidence. *Archives of Pathology and Laboratory Medicine*.

[49] Kleemann R., Verschuren L., Van Erk M. J. (2007). Atherosclerosis and liver inflammation induced by increased dietary cholesterol intake: a combined transcriptomics and metabolomics analysis. *Genome Biology*.

[50] Nistor A., Bulla A., Filip D. A., Radu A. (1987). The hyperlipidemic hamster as a model of experimental atherosclerosis. *Atherosclerosis*.

[51] Spady D. K., Dietschy J. M. (1988). Interaction of dietary cholesterol and triglycerides in the regulation of hepatic low density lipoprotein transport in the hamster. *Journal of Clinical Investigation*.

[52] Zhang Z., Wang H., Jiao R. (2009). Choosing hamsters but not rats as a model for studying plasma cholesterol-lowering activity of functional foods. *Molecular Nutrition & Food Research*.

[53] Gao S., He L., Ding Y., Liu G. (2010). Mechanisms underlying different responses of plasma triglyceride to high-fat diets in hamsters and mice: roles of hepatic MTP and triglyceride secretion. *Biochemical and Biophysical Research Communications*.

[54] Ali K. M., Wonnerth A., Huber K., Wojta J. (2012). Cardiovascular disease risk reduction by raising HDL cholesterol-current therapies and future opportunities. *British Journal of Pharmacology*.

[55] Libby P. (2000). Changing concepts of atherogenesis. *Journal of Internal Medicine*.

[56] Bentes M. H. S., Serruya H., Rocha Filho G. N., Oliveira Godoy R. L., Silva Cabral J. A., Soares Maia J. (1987). Estudo químico da semente do bacuri. *Acta Amazonica*.

[57] Fernandez M. L., West K. L. (2005). Mechanisms by which dietary fatty acids modulate plasma lipids. *Journal of Nutrition*.

[58] Miura T., Iwamoto N., komatsu Y. (2001). Hypolipidemic activity of mangiferin in cholesterol-fed mice. *Journal of Traditional Medicines*.

[59] Bao L., Hu L., Zhang Y., Wang Y. (2016). Hypolipidemic effects of flavonoids extracted from Lomatogonium rotatum. *Experimental and Therapeutic Medicine*.

[60] Jeppesen J. ø., Hein H. O., Suadicani P., Gyntelberg F. (1998). Triglyceride concentration and ischemic heart disease. *Circulation*.

[61] Basu M., Prasad R., Jayamurthy P., Pal K., Arumughan C., Sawhney R. C. (2007). Anti-atherogenic effects of seabuckthorn (hippophaea rhamnoides) seed oil. *Phytomedicine*.

[62] Martinello F., Soares S. M., Franco J. J. (2006). Hypolipemic and antioxidant activities from Tamarindus indica L. pulp fruit extract in hypercholesterolemic hamsters. *Food and Chemical Toxicology*.

[63] Lai Y.-S., Yang T.-C., Chang P.-Y. (2016). Electronegative LDL is linked to high-fat, high-cholesterol diet-induced nonalcoholic steatohepatitis in hamsters. *The Journal of Nutritional Biochemistry*.

[64] Yang T.-H., Yao H.-T., Chiang M.-T. (2017). Red algae (Gelidium amansii) hot-water extract ameliorates lipid metabolism in hamsters fed a high-fat diet. *Journal of Food and Drug Analysis*.

[65] Villachia H. (1996). *Frutales y Hortalizas Promisorios de Ia Amazonia*.

[66] Carvalho J. E. U. (2007). Aspectos botânicos, origem e distribuição geográfica do bacurizeiro. *Bacuri: Agrobiodiversidade*.

[67] Pesce C. (2009). *Oleaginosas da Amazonia, 2. Revisado. Belém: Museu Paraense Emílio Goeldi; Brasília*.

[68] Sabará D. N., Tibério J., Maria C. (2018). *Processo para produção de manteiga de bacuri refinada e clarificada, manteiga de bacuri refinada e clarificada, e seus usos cosméticos, farmacêuticos e nutracêuticos*.

[69] Fasciotti M., Monteiro T. V. C., Rocha W. F. C. (2020). Comprehensive triacylglycerol characterization of oils and butters of 15 amazonian oleaginous species by ESI‐HRMS/MS and comparison with common edible oils and fats. *European Journal of Lipid Science and Technology*.

[70] Yeung A. W. K., Souto E. B., Durazzo A. (2020). Big impact of nanoparticles: analysis of the most cited nanopharmaceuticals and nanonutraceuticals research. *Current Research in Biotechnology*.

